# Transcriptional Biomarkers and Immunohistochemistry for Detection of Illicit Dexamethasone Administration in Veal Calves

**DOI:** 10.3390/foods11121810

**Published:** 2022-06-20

**Authors:** Alessandro Benedetto, Elena Biasibetti, Elisa Robotti, Emilio Marengo, Valentina Audino, Elena Bozzetta, Marzia Pezzolato

**Affiliations:** 1 Istituto Zooprofilattico Sperimentale del Piemonte, Liguria e Valle d’Aosta, Via Bologna 148, 10154 Torino, Italy; alessandro.benedetto@izsto.it (A.B.); valentina.audino@izsto.it (V.A.); elena.bozzetta@izsto.it (E.B.); marzia.pezzolato@izsto.it (M.P.); 2 Department of Sciences and Technological Innovation, University of Piemonte Orientale, Viale Michel 11, 15121 Alessandria, Italy; emilio.marengo@uniupo.it

**Keywords:** gene expression, food safety, glucocorticoids, histology, muscle, Kohonen’s neural networks, PLS-DA, veal calves

## Abstract

Corticosteroids such as Dexamethasone (DEX) are commonly licensed for therapy in meat animals due to their known pharmacological properties. However, their misuse aimed to achieve anabolic effects is often found by National Residues Control Plans. The setup of a complementary “biomarker based” methods to unveil such illicit practices is encouraged by current European legislation. In this study, the combined use of molecular and histological quantitative techniques was applied on formalin fixed paraffin embedded (FFPE) muscle samples to assess the effects of illicit DEX treatment on veal calves. A PCR array, including 28 transcriptional biomarkers related to DEX exposure, was combined with a histochemical analysis of muscle fiber. An analysis based on unsupervised (PCA) and supervised (PLS-DA and Kohonen’s SOM) methods, was applied in order to define multivariate models able to classify animals suspected of illicit treatment by DEX. According to the conventional univariate approach, a not-significant reduction in type I fibres was recorded in the DEX-treated group, and only 12 out of 28 targeted genes maintained their expected differential expression, confirming the technical limitations of a quantitative analysis on FFPE samples. However, the multivariate models developed highlighted the possibility to establish complementary screening strategies, particularly when based on transcriptional biomarkers characterised by low expression profiles.

## 1. Introduction

Official controls on the residues of illicit growth promoters and other misused authorised drugs in meat products, both marketed within the European Community (EU) and imported from Third Countries, are strictly regulated to preserve consumer safety. Nevertheless, the illicit misuse of these compounds to increase animal production has still been detected by National Residues Control Plans (NRCPs) and Europol investigations, often revealing how easily steroids are accessed in illegal markets by easy-profit prone breeders.

Indeed, a broad range of illicit practices have been unveiled during the years by the cited control activities, but, unfortunately, similarly to sports doping, novel formulations based on both new designer drugs and already known performance enhancers are also continuously developed for farm animals [1,2]. Consequently, a constant update of new methods and techniques is needed, at both screening and confirmation levels.

In the list of misused substances found by NRCP surveys, authorised drugs such as corticosteroids are also often reported [3], being administered in illicit practices at low doses for long periods and beyond the expected withdrawal times, and/or in combination with other prohibited substances such as sex steroids, B2-agonists, etc. Among corticosteroids, Dexamethasone (DEX) is one of the most common drugs and is licensed for therapy in both humans and animals due to its known immunomodulation and anti-inflammatory properties.

In Italy, in 2008, the Italian Ministry of Health introduced the histological plan as a complementary strategy for the control of growth-promoting molecules misuse. The objective of this monitoring activity was to verify whether the samples from the slaughterhouses exceeded a predefined prevalence threshold level for illicit treatment with corticosteroids at the national level [4,5].

Indeed, as remarked by EFSA since 2013, the establishment of complementary diagnostic methods, based on biological effects and biomarkers of exposure to anabolic substances, could be useful to update current tests available in NRCPs [6]. In this context, the recent implementation of untargeted strategies, holistic approaches and “omics” technologies to unveil animal doping cases seems to be highly promising [1,7]. Moreover, the definition of panels of discriminant biomarkers, identified through different untargeted metabolomic, proteomic and transcriptomic analyses, could facilitate the establishment of cheaper and scalable diagnostic tests needed during field investigations [8,9,10].

Additionally, regarding DEX effects on cattle, different transcriptomics and proteomic studies have been proposed to firstly identify candidate biomarkers of exposure [11,12,13,14] and then validate their application in field samples [15,16]. However, the attempts to merge different sources of biological information to enhance the detection of such illicit practices, e.g., a combination of image analysis, immunohistology and gene expression studies, are currently limited to other classes of growth promoters such as sex steroids [17].

The aim of the work was therefore to combine the output of a Real Time PCR array, designed to quantify expression levels of multiple transcriptional biomarkers related to DEX exposure, with a histochemical measurement of muscle fibres. This coupled molecular/histological quantitative approach was applied on the same muscle samples that were formalin-fixed, paraffin-embedded (FFPE) and collected during an animal trial performed to assess the effects of illicit treatment with DEX on veal calves. The use of a multivariate analysis was tested to identify an optimal set of histological and/or transcriptional biomarkers that are able to classify animals suspected of illicit treatment by steroids, i.e., DEX.

## 2. Materials and Methods

### 2.1. Sample Selection

Archived paraffin blocks of bovine biceps brachii muscle samples, from a previous animal trial to study the effects of DEX illicit administration [18], were retrieved from the tissue bank of the National Reference Centre for Biological Investigation of Anabolic Substances in producing animals (CIBA).

Briefly, 2 month old Friesians male calves were recruited, randomly divided into two groups and reared for about 4 months. One month before slaughtering, one group was orally treated with dexamethasone 21-phosphate disodium salt at a dose of 0.4 mg/day per animal for 20 days, according to a presumed anabolic protocol of treatment, and the other group was kept as control and treated with a placebo. The animals were all slaughtered at about 7 months in an EC certified slaughterhouse approximately 10 days after the last drug administration; control animals were slaughtered after the treated ones. The experiment was carried out in accordance with the European Council Directive 2010/63/EU 86/609 (D.Lgs 26/2014) and was authorised by the Italian Ministry of Health and the Ethics Committee of the University of Turin. At the end of the sampling procedure, the carcasses of the treated animals were destroyed according to the relevant legislation (Directive 2003/74/EC). The study was a randomised controlled blind clinical trial. The biceps brachii muscle samples were collected at the slaughterhouse and immediately fixed in 10% neutral buffered formaldehyde and transferred to the laboratory.

Further and comprehensive information regarding the animal trial is available in the work of Richelmi et al., 2017 [18].

Forty FFPE muscle samples (15 controls + 25 DEX treated) from the cited animal trial were randomly chosen for microscopy and transcriptomics investigations, plus two additional FFPE samples from calves treated with a therapeutic dose of DEX. The authorised therapeutic schedule foreseen was dexamethasone 21-phosphate disodium salt (2 mg/kg of live weight for three consecutive days. These two samples were not included in model calculations (see Section 2.5) but were used for external prediction. A total of 42 FFPE samples was therefore considered in the study (see Section 3).

### 2.2. Histological Analysis

The samples were collected and oriented along the transverse fibre sectioning. Muscle samples were fixed in 10% neutral buffered formaldehyde, routinely processed, embedded in a paraffin box, sectioned in 3–5 μm slices and stained with haematoxylin and eosin (HE) to verify the correct orientation of the fibres. Only the samples correctly orientated and without alterations were selected for the morphometric analysis. The selected samples were then sectioned in 3–5 μm slices for the immunohistochemistry analysis. Sections were incubated in a buffer solution at pH 6 at 97 °C for 30 min to reduce the nonspecific binding of the secondary antibodies; then, they were incubated at room temperature in a humid chamber for 1 h with a 1:50 solution of a monoclonal antibody specific for the slow myosin heavy chain type 1 (MHC1) to detect type 1 fibres (MAB1628; Millipore, Burlington, VT, USA). After washing, the EnVision System Kit (Agilent Dako, Santa Clara, CA, USA) for polyclonal and monoclonal antibodies was used as the detection system. The immune reactions were visualised by applying a 3,3′-diaminobenzidine (DAB) chromogen solution (Dako) for 4′ (Agilent Dako, Santa Clara, CA, USA).

**Figure 1:**
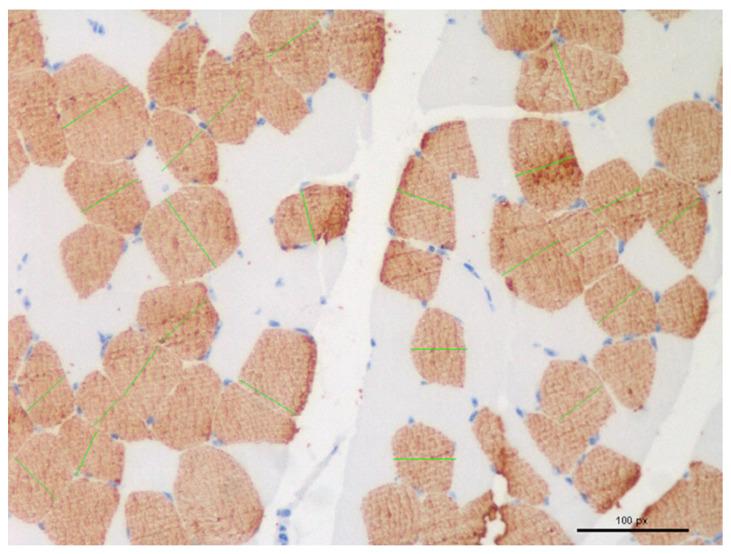
Selection and measurement (green lines) of muscle fibre for diameters calculations in randomly chosen fields.

All sections were analysed using optical microscopy and images were captured using a Nikon DS-Fi1 colour digital camera (Nikon Europe BV Instruments, Amsterdam, The Netherlands). For each slide, 25 muscle fibre diameters were measured after manual selection in 5 randomly selected 200× fields digital images (5 fibres in each image) (Figure 1). The NIS-Elements 4.5. software (Nikon Europe BV Instruments, Amsterdam, The Netherlands) was used to analyse the images. To overcome the distortion that may occur when a muscle fibre is cut obliquely, the “lesser diameter” was measured [19].

### 2.3. RNA Extraction

The total RNA was isolated from the same FFPE muscle samples used for the histological analysis and optimised procedures for long-term archived FFPE tissues were applied [20]. Briefly, 10 sections, of 8 µm thickness, were cut and processed with the miRNeasy FFPE kit (Qiagen, Düsseldorf, Germany), quantified with Qubit BR-RNA kit (Thermo Fisher Scientific, Waltham, MA, USA) and successively checked using Bioanalyzer 2100 (Agilent, Santa Clara, CA, USA) with the total RNA 6000 Nano kit (Agilent) for the RNA integrity number (RIN) and DV200 estimation.

### 2.4. Retro Transcription, Preamplification and Taqman Custom Arrays

A custom Taqman Real Time PCR Array (project number 4351372, Thermo Fisher Scientific) was designed on 96-wells optical plates to quantify the expression levels of the 28 genes that were identified in previous studies as related to the DEX exposure [11,14,15]. The Taqman assays for four reference genes, required for data normalisation (Table 1), were also spotted on the array. The chosen Real Time plate layout allowed for the analysis of each sample in triplicate in each PCR run (3 × 32 targets).

The total RNA from the extracted samples (2.5 µg) was reverse transcribed to a final volume of 20 µL with the SuperScript-IV Vilo kit (Thermo Fisher Scientific). The cDNA from each sample was then preamplified with Taqman Preamp Master Mix and Custom preamp primers pool (Thermo Fisher Scientific) according the manufacturer’s guidelines, which were as follows: hold stage of 10 min at 95 °C, 14 cycles of 15 s at 95 °C and 4 min at 60 °C, followed by final denaturation and Taq polymerase inactivation step (99 °C for 10 min). The conclusive Real Time PCR step on preamplified cDNAs (tenfold diluted in TE buffer) was performed using the Taqman Fast Advance Master Mix (Thermo Fisher Scientific) and a StepOne Plus Real-Time PCR System (Thermo Fisher Scientific) with the following thermal cycling conditions (fast ramp mode): two consecutive holding stages (2 min at 50 °C and 2 min at 95 °C), followed by 40 cycles of 1 s at 95 °C and 20 s at 60 °C (final reaction volume in each well: 10 µL).

### 2.5. Data Analysis

The collected histological data were analysed using Prism software 6.01 (GraphPad software, Inc. San Diego, CA, USA). The Shapiro–Wilk test was used to test the normality of the data distribution before the statistical analyses. The data were described by mean and standard deviation (SD). Morphometry analysis-related differences were also assessed by unpaired *t*-test and *p*-values < 0.05 were considered statistically significant. Relative quantification (RQ) of the 28 selected transcripts was performed using the ∆∆Cq method [21]. Preliminary analysis and filtering of Cq values was performed with the GeneEx 6.1 software (MultiID Analyses AB, Göteborg, Sweden), by the evaluation of mean, standard deviation, and outlier identification. Specifically, a threshold value of 32 Cq was applied to all the collected gene expression data, as suggested by the Taqman Array supplier (Thermo Fisher Scientific) when the cDNA preamplification step was performed. To evaluate the stability of the reference genes, Genorm and Normfinder analyses were applied [22,23].

The fold changes of all biomarkers for both the control and treated groups of muscle samples were reported using the log2 scale ± confidence interval (CI) at 95%. The significance of recorded fold changes was assessed by one-way ANOVA with Tukey’s post hoc test (* *p* < 0.05).

A multivariate statistical approach was then applied to all the collected data, consisting of a pattern recognition approach, followed by a classification analysis, to verify the ability of the measured variables in classifying control vs. DEX-treated samples. The analysis was carried out on the following two datasets:

− RQ dataset, consisting of 52 gene expression profiles described by 30 variables each, achieved from the analysis of 37 samples (see Section 3) plus several randomly chosen technical replicates at different dilution levels, in order to test and exclude a significant quantification bias due to the cDNA preamplification step, as described by Korenková et al. [24]. This whole dataset comprised 12 controls and 23 treated samples, plus two more samples from animals treated with a therapeutic dose of dexamethasone. These two samples were used for external prediction and were not included in the model calculations;
− RQhisto dataset, including the RQ variables recorded by the gene expression analysis and the results obtained by the histologic analysis, i.e., the average, the median and the standard deviation of the measurements taken for each sample. The total number of analysed samples in this hybrid genomic and microscopic dataset was lower than in the genomic only (RQ) dataset, since suitable and well-oriented muscular fibres from the respective paraffin-embedded blocks used for the microscopy analysis with complete gene expression profiles were not available for all samples. The final RQhisto dataset therefore consisted of 35 samples described by 33 variables. This dataset comprised 12 controls, 22 treated samples and just 1 sample treated with a therapeutic dose of dexamethasone, that was exploited for external prediction and was not included in the model calculation.

Gene expression data in both datasets were firstly normalised according to Genorm and NormFinder results, then treated using Principal Component Analysis [25], Partial Least Squares—Discriminant Analysis (PLS-DA) [8,25,26] and Supervised Kohonen’s Self Organising maps [27].

#### 2.5.1. Principal Component Analysis (PCA)

PCA [25] is a multivariate pattern recognition method which provides a new set of orthogonal variables called Principal Components (PC), obtained as linear combinations of the original variables. PCA provides the following two tools for data analysis: the scores, i.e., the projections of the samples on the space given by the PCs, and the loadings, i.e., the coefficients of each variable in the linear combination describing each PC. Both scores and loadings can be analysed graphically by representing them on the space given by two PCs at a time, to identify groups of samples with a similar behavior (score plot) and the reasons for the observed grouping and correlations between the variables (loading plot). More details can be found in [25].

#### 2.5.2. Partial Least Squares Discriminant Analysis (PLS-DA)

Partial Least Squares (PLS) [8,25,26] is a multivariate regression method correlating a series of descriptors (X variables) to one or more experimental responses (Y variables): the method searches for latent variables (LVs), similar to principal components, built on the X variables which mostly correlate to LVs calculated on the Y variable(s). PLS-DA, a modification of PLS, is devoted to classification purposes. The classification performances were evaluated on the basis of several parameters, which are as follows: accuracy %, Non-Error-Rate % (NER %), sensitivity, specificity and precision [28]. Here, PLS-DA was applied to the RQ dataset and RQhisto dataset independently, after autoscaling. The results were calculated both in fitting and in cross-validation, eliminating at each iteration 20% of all the samples (1000 iterations).

#### 2.5.3. Kohonen’s Self Organizing Maps (Kohonen’s SOMs)

Kohonen’s SOMs [27] are artificial neural networks capable of solving complex problems by simulating the functioning of the human brain. They are based on an auto-associative unsupervised algorithm, whereby the input data are presented to the network which groups them depending on their similarity. This similarity can be general (based on all the variables) or local (based on a subset of the variables employed to describe the problem). Kohonen’s SOMs are based on a single layer of neurons, usually arranged in a square (top layer) where the samples appear grouped at the end of the learning phase. Below each neuron of the top layer, there is a column of cells, one for each descriptor (X variables, here the signals), which contain the weights of the network. During each learning epoch, every sample is presented in turn to the network. For each sample, the distance between the sample and every column of weights is calculated. The column with the minimum distance is considered as the winning neuron. The weights of this neuron are modified so that at the subsequent cycle, the distance of the same sample from the winning neuron is the smallest. A similar correction is applied to the neurons in the neighbourhood of the winner but the correction decreases with the distance and usually also decreases with the number of epochs. At the beginning, the whole network is affected by the corrections while in the last cycles only the winning neuron is corrected. Similarly, at the beginning, the learning rate, i.e., the amount of correction introduced, is larger than in the last cycles. The aim of Kohonen learning is to map similar signals to similar neuron positions. The final result is a map of neurons, where the most similar samples are in the same cell or in close cells. The weights below each neuron provide insight into the reason for the clusterisation of the objects.

Kohonen’s networks can also be trained in a supervised manner to provide sample classification (Supervised Kohonen networks—SKN) [27], whereby the input (X) map and the output (Y) map are combined in an input–output map that is trained in the same way standard Kohonen’s maps. Here, SKN was run with the following settings: toroidal boundary, batch algorithm, hexagonal topology, random initialization of weights, learning rate decreasing linearly from 0.5 to 0.01, a top map with 8 × 8 neurons and 200 training epochs.

### 2.6. Software for Multivariate Data Analysis

PCA and PLS-DA were carried out with Matlab R2014a (The Mathworks, Natick, MA, USA), utilizing the in-house-developed routines and the Classification Toolbox from Milano Chemometrics [28]; Kohonen SOMs were built with the Kohonen and CP-ANN (Counter propagation artificial neural network) toolbox for MATLAB from Milano Chemometrics [29]. Graphical representations were carried out using Matlab, Statistica v.7 (Statsoft Inc., Tulsa, OK, USA) and Excel 2016 (Microsoft Corporation, Redmond, WA, USA).

## 3. Results

### 3.1. Histological Analysis

Only thirty five out of forty two samples selected for the gene expression study were also suitable for the morphometry analysis, four samples showed an incorrect fibre orientation, and one further sample was excluded since it presented eosinophilic myositis. No significant differences were identified between the diameter measure of the fibres in the canonical univariate analysis (*p* > 0.05). Descriptive statistics of the morphometry analysis are reported in Table 2.

### 3.2. Gene Expression Analysis

The RNA extraction was correctly performed on 37 of 42 FFPE samples from the animal trial. The RIN number of all extracts ranged between 2.1 and 5.4 and DV200 scores of all samples were between 30% and 50%, confirming the high fragmentation of RNA extracted from FFPE muscle samples. This evidence was further validated by the amplification profiles collected for the 32 targeted genes: four targets (OXT, GALNT9, FISIP1 and GAD1) in some samples had Cq values over the cut-off for pre-amplified cDNA (>32 Cq).

Genorm and NormFinder analyses revealed RPLP0 and HSP8A as the most stable genes for data normalisation.

**Figure 2:**
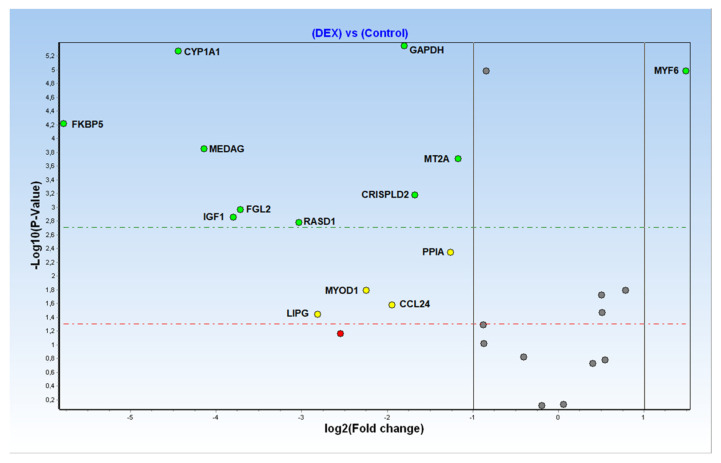
Volcano plot of Fold Changes (X axis, logarithmic scale) and related *p*-values (Y axis, logarithmic scale) for all considered transcriptional biomarkers.

The differentially expressed genes (DEG), defined by the comparison of the normalised expression levels from DEX-treated and control groups, are reported with the associated *p*-values in Figure 2.

**Figure 3:**
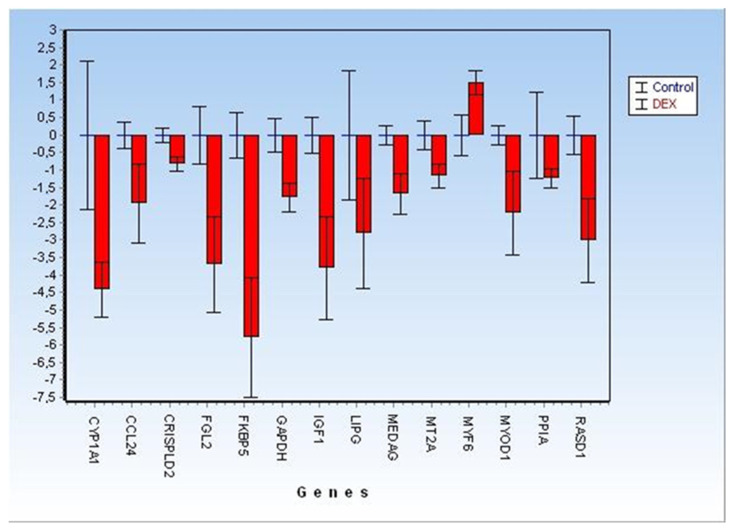
Detailed Fold Changes (±CI at 95%) of 12 significant differentially expressed genes (represented as green and yellow dots, respectively, in Figure 2) plus two targets (PPIA and GAPDH) analysed for reference-genes selection.

Of the 28 biomarkers originally selected for array design, only 12 targets plus two genes tested as reference genes were found to be still differentially expressed (DE) in the analysed FFPE muscle samples (Figure 3).

### 3.3. Multivariate Analysis

#### 3.3.1. PCA

**Figure 4:**
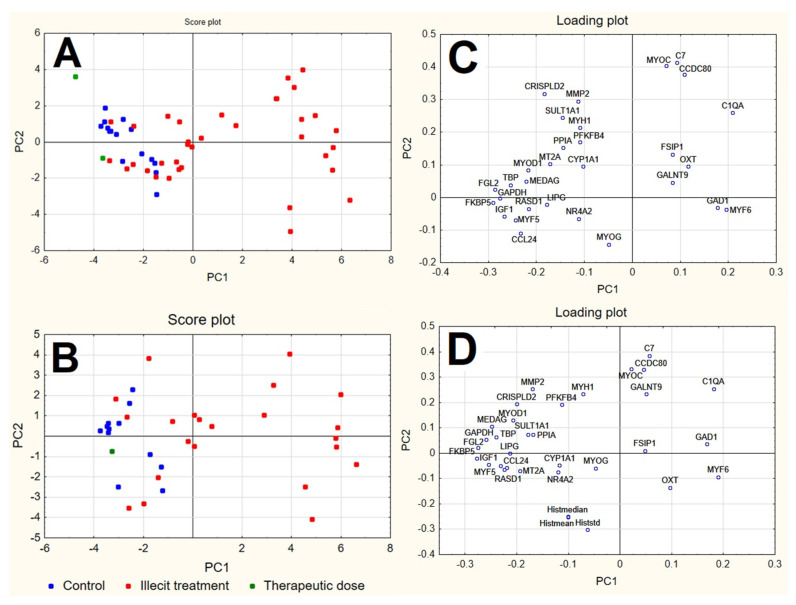
Score plot and loading plot of the first two PCs calculated for RQ dataset (**A**,**C**) and RQhisto dataset (**B**,**D**), after autoscaling.

PCA was applied to both the RQ and RQhisto datasets after autoscaling (mean centring followed by unit variance normalisation). In both cases, the first four PCs explain more than 60% of the overall information (see Supplementary Material Table S1), showing a considerably correlated data structure. PCA shows in both cases that the samples separated along PC1, with controls having negative scores and treated samples demonstrating more positive scores along the first PC. The samples treated by the therapeutic dose appeared within the group of controls (Figure 4).

Looking at the corresponding loading plots, both datasets reveal variables at positive loadings on PC1 (e.g., C1QA, GAD1, MYF6) to be characterised by larger signals in illicitly treated samples and lower signals in controls, while variables at negative loadings on PC1 (e.g., FGL2, KKBP5, GAPDH, IGF1, MYF5, CCL24, TBP, MYOD1, MEDAG, RASD1) show an opposite behavior. The subsequent PCs do not show a clear separation of the samples into groups and are not shown.

#### 3.3.2. PLS-DA

PCA highlighted the possibility of identifying the two groups of samples (controls and DEX treated ones) by means of the most significant PCs in an unsupervised approach. PLS-DA was therefore applied to both RQ and RQhisto datasets separately to identify biomarkers of the illicit treatment with a supervised approach. Notwithstanding the reduced size of the datasets, the supervised approach, coupled to cross-validation procedures, was able to identify the candidate biomarkers more reliably.

PLS-DA was therefore applied on both datasets, using a cross-validation procedure with the elimination of 20% of the samples from the training set at each validation step, with 1000 repetitions. For both datasets, the best results in cross-validation were obtained with 2 LVs in the final model. In both cases, the class of the samples corresponding to the therapeutic dose were not included in model calculations but were exploited for an external prediction.

The two models show satisfactory performances in fitting (NER% about 90%) and a slightly decreased level of performance in cross-validation (about 86–88%) (Table 3) according to the NER%, the accuracy and the values of precision, specificity and sensitivity calculated for the two classes. The misclassifications were six for the RQ dataset and four for the RQhisto dataset. In both cases, the misclassifications were within the illicit treatment class. In particular, of the six samples misclassified by PLS-DA applied to the RQ dataset, four were the same specimens wrongly classified as untreated samples in the RQhisto dataset analysis.

**Figure 5:**
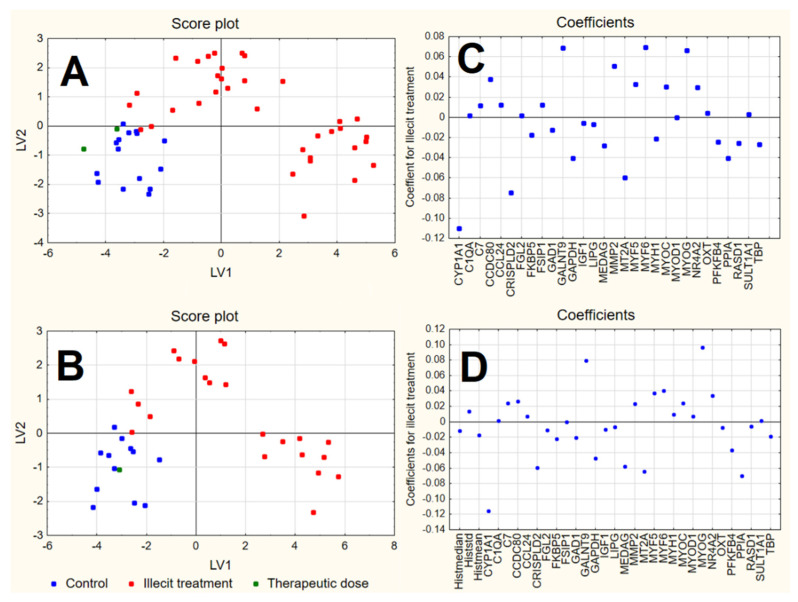
Score plot and plot of the coefficients of the first 2 LVs calculated by PLS-DA on RQ (**A**,**C**) and RQhisto (**B**,**D**) datasets.

The score plot of the first two LVs (Figure 5) shows that, for both datasets, control samples were quite well separated from the illicitly treated samples, by means of the first two LVs, with controls expressing negative scores on both LVs and the illicitly treated samples divided into the following two groups: one with positive scores on LV2 and the other with positive scores on LV1.

The samples characterised by a therapeutic dose, not included in the model calculations, were predicted by both models as belonging to the control class. In both cases, positive coefficients correspond to variables with higher values in samples treated by illicit doses of dexamethasone and lower values in control samples; however, variables characterised by negative coefficients show instead an opposite behaviour. The plot of the coefficients for the RQhisto dataset reveals that the three variables related to histology do not show high coefficients and seem not to play a relevant role in the final model. Among the variables characterised by a relevant coefficient in the final models, CYP1A1, CRISPLD2, MT2A, GAPDH and PPIA showed the highest negative coefficients and therefore appear to be more expressed in the control samples, while GALNT9, MYOG, MYF6 and MMP2 showed the highest positive coefficients and were therefore more expressed in the illicit treatment. The only difference between the two models consists of the variables characterised by the highest positive coefficients that, in the case of the RQhisto dataset, do not include MYF6 and MMP2.

#### 3.3.3. Supervised Kohonen’s Self Organising Maps

Kohonen’s supervised SOMs were then applied to both datasets separately, with the following setup: a top map of 8 × 8 hexagonal neurons, toroidal boundary, batch algorithm, random initialization of weights, learning rate decreasing linearly from 0.5 to 0.01 and 200 training epochs [27]. The data were autoscaled prior to range scaling. The results in fitting and cross-validation (20% of the samples eliminated from the training set at each iteration, 1000 iterations) for both datasets are given in Table 4.

For both datasets, the results in fitting are better than PLS-DA, thereby providing the perfect classification of all the samples, while the models show acceptable predictive abilities in cross-validation, slightly better than PLS-DA. In both cases, the samples with therapeutic doses were predicted in the control class.

**Figure 6:**
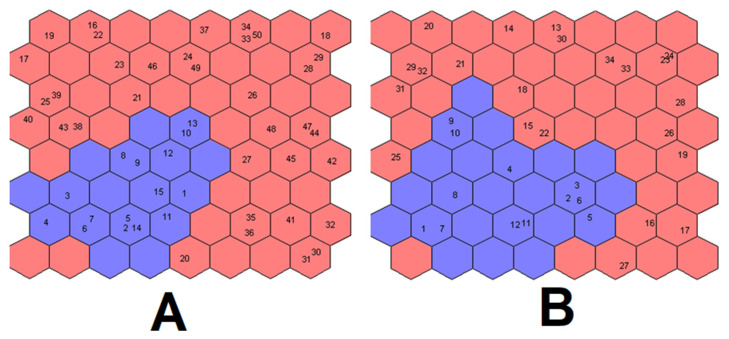
Kohonen’s top map obtained for RQ dataset (**A**) and RQhisto dataset (**B**). Blue neurons are attributed to the control class, while red neurons to the dexamethasone-treated class.

The top maps obtained for both datasets are represented in Figure 6. In both cases, the two classes appear well separated from each other: similar samples are contained in the same neuron or in nearby neurons on the top map.

**Figure 7:**
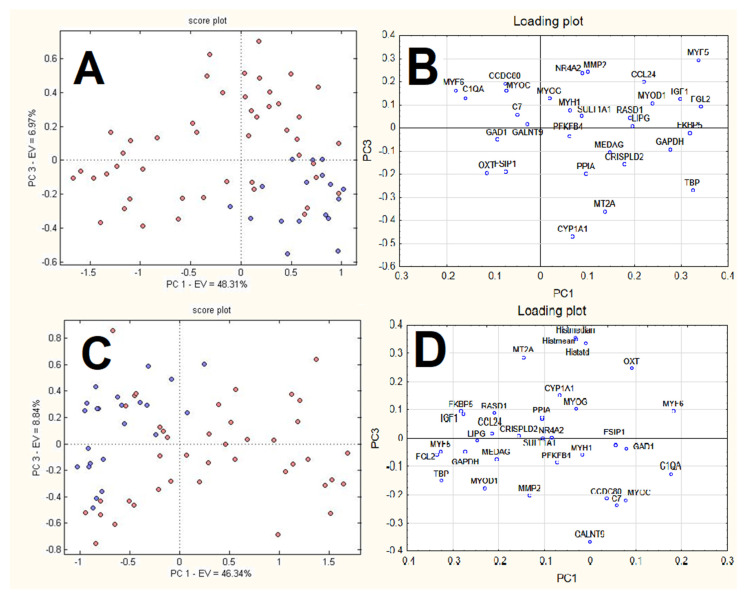
Results of PCA applied to the weights calculated for each neuron of the top map and each variable: score (**A**) and loading plot (**B**) for RQ dataset; score (**C**) and loading (**D**) for RQhisto dataset. The score plots report the neurons of the top map in different colours: blue circles correspond to neurons attributed to controls while red circles correspond to neurons attributed to dexamethasone-treated samples.

The results of the PCA applied to the weights calculated for each neuron of the top map and each variable for RQ dataset and RQhisto dataset, respectively, are reported in Figure 7. The score plot reports the neurons of the top map in different colours, where blue circles correspond to neurons attributed to controls while red circles correspond to neurons attributed to dexamethasone-treated samples. The first and the third PCs appear in both cases to be the best ones to separate the two groups of neurons. The corresponding loading plots report the weights of the original variables in the space given by the same PCs. For the RQ dataset (Figure 7A,B), control neurons have positive scores for PC1 and negative ones for PC3. These samples show high signals for variables MT2A, TBP, GAPDH, CRISPLD2, PPIA, CYP1A1, MEDAG, and low signals for variables MYF6, CCDC80, MYOC, 1QA, GAD1, C7, GALNT9; however, dexamethasone-treated samples show an opposite behaviour.

For the RQhisto dataset (Figure 7C,D), control neurons have negative scores on PC1 and positive ones on PC3. These samples show high signals for variables MT2A, MYF5, PGL2, FKBP5, CYP1A1, RASD1, PPIA, CCL24, GAPDH and small signals for variables MYF6, C1QA, OXT, GAD1, FSIR1, CCDC80, MYOC; however, dexamethasone-treated samples show an opposite behaviour.

## 4. Discussion

The detection and quantification of transcriptional biomarkers in FFPE samples is known to be biased by the fragmentation and degradation grade of nucleic acids induced by formalin [30]. Moreover, the profiling of transcripts characterised by low basal expression levels and/or strong down-regulation induced by the investigated illicit treatment could add further confounding variance to the quantitative analysis, overcoming in some cases the sensitivity of PCR-based methods (e.g., late Cq values, higher SD, etc.). Consequently, the reproducibility of the applied molecular methods needs to be carefully assessed through the application of sufficient technical and biological replicates [31].

In our study, the choice of as-short-as-possible amplification regions for all the considered transcripts (see Table 1) and cDNA preamplification strategies was therefore adopted by testing different dilutions of the preamplified samples, in order to obtain the maximum possible consistency of the recorded quantification data, following previous experience of profiling multiple transcriptional biomarkers in FFPE liver samples [8]. The use of more expensive Taqman chemistry instead of cheaper fluorescent dsDNA dyes-based assays, combined with cDNA preamplification, led to successful gene-expression profiling in FFPE muscle samples.

However, a lack of reproducibility, late Cq and/or no amplification was observed in some of the analysed samples, especially for the following targets: OXT, GALNT9, FISIP1 and GAD1. Therefore, according to the conventional univariate approach, only 12 of the analysed targets maintained significant (*p* < 0.05) differential expression levels (Figure 2 and Figure 3), being characterised by a broader CI in comparison with the fold changes described by previous studies on fresh/frozen or RNAlater muscle tissues [11,13,14,15].

Therefore, with the application of different multivariate approaches, namely pattern recognition analysis (PCA) and classification methods (PLS-DA and Kohonen’s supervised SOMs), all the biological information coming from the whole RQ dataset was considered, providing a promising classification of the untreated (control) and DEX therapeutic treated samples (predicted by both models as belonging to the control group), with only a few misclassified samples. In particular, during cross-validation with Kohonen’s SOMs, six samples appeared to be misclassified in the group of anabolic DEX-treated samples, while the fitting of the models was perfect.

The application of PLS-DA helped to identify the candidate biomarkers in a more reliable way with respect to PCA, through the exploitation of a supervised approach coupled to cross-validation strategies which helped in the identification of candidate biomarkers. This is also highlighted by the fact that some of the variables with significant loadings on PC1 in the PCA analysis (e.g., GAD1, C1QA shown in Figure 4) were not characterised by a very high coefficient in the PLS-DA models (Figure 5); the significant LVs in PLS-DA did not signify the same results as PCs in PCA, highlighting that the two methods achieve different goals, i.e., the directions of maximum variance identified by PCA do not completely correspond to the most discriminant directions identified by PLS-DA.

Comparing the results of PLS-DA and supervised Kohonen’s SOMs, the classification performances reached by Kohonen’s SOMs indicate better results both in fitting and in cross-validation, particularly for the accuracy of classification and the parameters calculated for each class (Precision, Sensitivity, Specificity). For the variables characterised as discriminating, a certain overlapping between the two methods was observed, since the variables identified as the most relevant in PLS-DA (those with the largest absolute value of the coefficient) were also identified as relevant by Kohonen’s SOMs; however, ANNs (Artificial neural networks) identified some additional variables as relevant for classification, e.g., CCL24, GAPDH, RASD1, FGL2, MYOD1, MEDAG, FKBP5.

The applied tools were indeed limited by the reduced number of analysed samples; consequently, to increase the prediction capabilities of the described multivariate models, additional biological information on the analysed samples was added by means of the histological characterisation of the muscle fibres, for which it is known that adaptive changes can occur in response to variations in the pattern of neural stimulation, loading conditions, availability of substrates, and hormonal signals [32].

Our results indicate a not-significant reduction in type I fibres; these data can be justified because it was previously reported that type 2 muscle fibres are more pronounced than type 1 fibres due to a chronic exposure to excess glucocorticoid [32].

The choice to study only type 1 fibres was made because the biceps brachii of bovines contains a large percentage of type I myofibers [33]. Moreover, immunohistochemical studies were performed on paraffin-embedded sections, normally used only to evaluate inflammation and to identify the morphology of invading inflammatory cells [34]. This technical aspect may have influenced the results.

Accordingly, the fusion of different datasets (transcriptional and histological biomarkers), for the same DEX-treated and control FFPE muscle samples, revealed only a small improvement in the developed models in terms of fitting and cross-validation performances, with a slight reduction in the number of misclassified samples (from six to four samples) when RQ and RQhisto dataset performances were compared (Table 3 and Table 4). However, this first attempt to join different layers of biological information (mRNA levels and morphometric features on the same FFPE blocks) underscores the potential of a more extensive chemical description of the system, as already reported by other authors in different research fields [35]. Further evaluations on larger sample sets are therefore necessary to verify the performances of the tested models.

## 5. Conclusions

The present study confirmed that a gene expression analysis can be successfully applied on FFPE muscle samples, and that it can, in the future, be used to update the current Italian histological NRCP, to contrast both the illicit administration of unauthorised drugs (sex steroids, Beta-Agonists, etc.) and misuse of authorised drugs such as corticosteroids [4]. The multivariate approach revealed, once again, the possibility to set up complementary screening strategies similarly to what has already been developed for other food commodities [8], to obtain more biological information for sample classification in comparison to the common univariate approach and to promote the reliable profiling of transcriptional biomarkers when characterised by “challenging to quantify” low expression levels. The choice of muscular tissues could also help to develop novel control activities for third-country imported meat in the future, where the conventional sampling of target organs and biological fluids for illicit growth-promoters detection (gonads, sexual accessory glands, thymus, thyroids, liver, urine, blood, etc.) is often not possible.

## Figures and Tables

**Table 1:** List of selected targets for Gene expression study, reference sequences, amplicon sizes and supplier references for each Taqman assay. Reference gene for data normalisation marked with *.

Taqman Assay ID	Gene	Ref. Seq	Amplicon Size
Bt03218086_m1	RPLP0 *	NM_001012682.1	71 bp
Bt03238680_m1	FGL2	NM_001046097.1	93 bp
Bt03241948_m1	TBP *	NM_001075742.1	87 bp
Bt03238185_m1	C7	NM_001045966.1	59 bp
Bt03262383_m1	CCDC80	NM_001098982.2	63 bp
Bt03263026_m1	CRISPLD2	NM_001100299.1	55 bp
Bt04298466_m1	FKBP5	NM_001192862.1	65 bp
Bt03216009_m1	MMP2	NM_174745.2	65 bp
Bt03230953_m1	MYOC	NM_174118.2	74 bp
Bt04318503_g1	RASD1	NM_001206261.2	107 bp
Bt03217338_m1	SULT1A1	NM_177521.2	73 bp
Bt07108870_s1	CYP1A1	XM_002696635.5	129 bp
Bt03235950_m1	CCL24	NM_001046596.2	69 bp
Bt04298185_m1	PFKFB4	NM_001192835.1	69 bp
Bt03218751_m1	C1QA	NM_001014945.2	65 bp
Bt03210913_g1	GAPDH *	NM_001034034.2	66 bp
Bt03230996_g1	HSPA8	NM_174345.4	83 bp
Bt03257092_m1	MEDAG	NM_001083660.1	67 bp
Bt03217196_g1	OXT	NM_176855.1	113 bp
Bt03224615_g1	PPIA *	NM_178320.2	76 bp
Bt03252282_m1	IGF1	NM_001077828.1	65 bp
Bt03223166_m1	MYH1	NM_174117.1	74 bp
Bt03244740_m1	MYOD1	NM_001040478.2	84 bp
Bt03258929_m1	MYOG	NM_001111325.1	85 bp
Bt03223133_m1	MYF5	NM_174116.1	77 bp
Bt03224711_g1	MYF6	NM_181811.2	57 bp
Bt03248872_m1	NR4A2	NM_001076208.1	60 bp
Bt04317546_g1	MT2A	NM_001075140.1	87 bp
Bt04315715_m1	FSIP1	NM_001193138.2	69 bp
Bt03256973_m1	GALNT9	NM_001083641.1	59 bp
Bt03247326_m1	GAD1	NM_001075756.2	57 bp
AR2W94D	LIPG	XM_002697766.5	115 bp

**Table 2:** Descriptive statistics of morphometry analysis performed on 34 samples (22 from DEX illicit treated animals, 12 from controls). Data of the only sample from an animal treated with therapeutic dose of dexamethasone are not reported.

Parameters	DEX	Control
Samples	22	12
Mean	40.5	44.6
SD	9.0	7.5
SEM	2.1	2.5
Minimum	23.6	33.3
Median	39.1	46.4
Maximum	54.8	59.9

**Table 3:** Classification performances in fitting and cross-validation for PLS-DA applied to the RQ dataset (a) and to the RQhisto dataset (b), reporting Non-Error-Rate % (NER), Accuracy %, Sensitivity, Specificity and Precision.

Datasets	Model	NER%	Accuracy%	Groups	Precision	Sensitivity	Specificity
**RQ**	Fitting	91.43	88.00	Control	71.43	100	82.86
				Dex	100	82.86	100
	Cross-validation	89.91	84.91	Control	66.24	100	78.56
				Dex	100	78.56	100
**RQhisto**	Fitting	90.91	88.24	Control	75.00	100	81.82
				Dex	100	81.82	100
	Cross-validation	86.93	83.71	Control	69.13	98.12	75.74
				Dex	98.64	75.74	98.12

**Table 4:** Classification performances in fitting and cross-validation for Supervised Kohonen Networks applied to the RQ dataset (a) and to the RQhisto dataset (b), reporting Non-Error-Rate % (NER), Accuracy %, Sensitivity, Specificity and Precision.

Datasets	Model	NER %	Accuracy %	Groups	Precision	Sensitivity	Specificity
**RQ**	Fitting	100	100	Control	100	100	100
				Dex	100	100	100
	Cross-validation	87.67	88.59	Control	78.49	85.37	89.97
				Dex	93.48	89.97	85.37
**RQhisto**	Fitting	100	100	Control	100	100	100
				Dex	100	100	100
	Cross-validation	87.46	87.72	Control	78.64	86.70	88.23
				Dex	92.99	88.23	86.70

## Data Availability

The data presented in this study are available on request from the corresponding author. The data are not publicly available due to privacy.

## Supplementary Materials

The following supporting information can be downloaded at: https://www.mdpi.com/article/10.3390/foods11121810/s1, Table S1: Rates (%) of explained and cumulative explained variance by the first six PCs calculated for RQ dataset and RQhisto dataset.
