# Supplementary material for: Transcriptional Biomarkers and Immunohistochemistry for Detection of Illicit Dexamethasone Administration in Veal Calves
Source: Foods. 2022 Jun 20;11(12):1810. doi: 10.3390/foods11121810 (PMC9222442; doi:10.3390/foods11121810)
Supplement: Supplementary file 1 [file foods-11-01810-s001.zip › foods-1741011-supplementary.pdf]

Supplementary Material Table S1: Rates (%) of explained and cumulative explained variance by the first six PCs calculated for RQ dataset and RQhisto dataset.

	**RQ dataset**		**RQhisto dataset**	
	**% Expl. Var.**	**% Cum. Expl. Var.**	**% Expl. Var.**	**% Cum. Expl. Var.**
**PC1**	35.54	35.54	35.88	35.88
**PC2**	11.26	46.81	11.46	47.34
**PC3**	7.05	53.86	9.44	56.78
**PC4**	6.85	60.71	7.42	64.19
**PC5**	5.36	66.08	5.18	69.38
**PC6**	4.05	70.13	4.27	73.65
